# Gentamicin Induced Nephrotoxicity: The Role of Sex Hormones in Gonadectomized Male and Female Rats

**DOI:** 10.1155/2016/5025097

**Published:** 2016-04-26

**Authors:** Fatemeh Eshraghi-Jazi, Ardeshir Talebi, Fatemeh Sadat Mirsaeedi, Sarina Ahmadian, Fatemeh Moslemi, Mehdi Nematbakhsh

**Affiliations:** ^1^Water and Electrolytes Research Center, Isfahan University of Medical Sciences, Isfahan 81745, Iran; ^2^Department of Clinical Pathology, Isfahan University of Medical Sciences, Isfahan 81745, Iran; ^3^Department of Physiology, Isfahan University of Medical Sciences, Isfahan 81745, Iran; ^4^Isfahan-MN Institute of Basic and Applied Sciences Research, Isfahan 81546, Iran

## Abstract

*Background*. Gentamicin (GM) induced nephrotoxicity may be sex hormones related. The effects of sex hormones on GM induced nephrotoxicity in gonadectomized rats were investigated.* Methods*. Ovariectomized rats received 0.25, 0.5, or 1 mg/kg/week of estradiol (ES) alone or accompanied with 10 mg/kg/week of progesterone (Pro) for two weeks followed by GM (100 mg/kg/day) for 9 days. Castrated rats were also treated with 10, 50, or 100 mg/kg/week of testosterone (TS) for two weeks and then received GM. In addition, a single castrated group received 0.25 mg/kg/week of ES plus GM.* Results*. GM increased the serum levels of blood urea nitrogen (BUN) and creatinine (Cr) and kidney tissue damage score (KTDS) (*P* < 0.05). TS had no effect on the serum levels of BUN and Cr and KTDS, while low dose of ES intensified these parameters in male (*P* < 0.05). ES (0.5 mg/kg) without Pro ameliorated KTDS in female (*P* < 0.05) while ES (1 mg/kg) with or without Pro exacerbated the BUN values and Cr values, KTDS, and body weight loss (*P* < 0.05).* Conclusion*. ES (0.5 mg/kg) without Pro ameliorated kidney damage induced by GM in female while neither TS nor ES had beneficial effect on nephrotoxicity induced by GM in male, although ES aggravated it.

## 1. Introduction

Gentamicin (GM) is one of the aminoglycoside drugs which is commonly used for treatment of negative gram bacterial infections [1, 2]. The most important side effect of this drug is nephrotoxicity [3] which is accompanied with elevating blood urea nitrogen (BUN) and creatinine (Cr) levels in serum [4, 5]. GM also disturbs hemodynamic condition of kidney [6] and increases generation of reactive oxygen species by renal cortical mitochondria [7]. It also develops apoptosis in renal cortex [8].

Gender difference impresses prevalence and progression of kidney diseases [9, 10]. Some studies showed the impact of gender in cisplatin induced nephrotoxicity model [11–15] while GM induced gender related difference in some biomarkers [16]. Accordingly it seems that sex hormones play an important role in GM induced nephrotoxicity. Several studies reported various effects of estradiol (ES) and testosterone (TS) in nephrotoxicity induced by cisplatin and GM [17–23]; however, all the dimensions are not well understood. In addition, estrogen and progesterone (Pro) inhibit apoptosis [24]. The effects of either ES alone or the combination of ES and Pro in nephrectomy model were revealed [25]. This study was designed to investigate the effect of Pro and ES on nephrotoxicity induced by GM in ovariectomized rats. In addition, the role of TS and ES on GM induced nephrotoxicity was studied in castrated rats.

## 2. Materials and Methods

### 2.1. Animals

52 female (190.8 ± 2.8 g) and 27 male (210.6 ± 3.0 g) Wistar rats were used. The animals were housed in standard conditions and 12 h light/12 h dark cycle accompanied with free access to water and food. This experiment was approved in advance by the Isfahan University of Medical Sciences Ethics Committee.

### 2.2. Study Design

Male and female rats were gonadectomized [19, 20]. After one week as recovery time, the ovariectomized animals were divided into 9 groups. Groups 1 (*n* = 6) and 2 (*n* = 5) received sesame oil, and groups 3–5 (*n* = 5, 5, and 6) received 0.25, 0.5, or 1 mg/kg/week i.m. ES dissolved in sesame oil for a period of two weeks, respectively. Then groups 2–5 were treated with GM (100 mg/kg/day; i.p.) for 9 days while group 1 as control received saline instead of GM. Groups 6–9 (*n* = 6, 6, 7, and 6) followed the same regimen as groups 2–5 but Pro (10 mg/kg/week; i.m.) was added to regimen for the first two weeks.

Castrated rats were divided into groups 10–15 (*n* = 4, 5, 4, 4, 5, and 5). Groups 10–14 received the regimen similar to groups 1–5, but with TS (10, 50, and 100 mg/kg/week; i.m.) instead of different doses of ES, respectively. In addition, group 15 had the same regimen as group 3 in female groups. The summarized assigned groups are tabulated in Table 1. Four hours after last injection of GM in the 9th day, all the animals were anesthetized and blood samples were taken by heart puncture. The animals were sacrificed and kidney and uterus were removed and weighed immediately. Left kidney was fixed in formalin 10% for histopathological investigation. Right kidney was homogenized and centrifuged. Then both supernatant and serum samples were kept in −20°C until measurement.

### 2.3. Measurements

The serum levels of Cr and BUN were determined by quantitative diagnostic kits (Pars Azmoon, Iran) using automatic analyzer (Technicon, model RA1000). Malondialdehyde (MDA) level was quantified by a manual method. At first a solution was prepared including 15 g trichloroacetic acid, 0.375 g thiobarbituric acid, and 2 mL hydrochloric acid in total volume of 100 mL. Then 2 mL of the prepared solution and 1 mL of sample were mixed. The mixture was incubated in boiling water bath at the temperature of 100°C for 60 minutes and, after cooling, the mixture was centrifuged for 10 minutes. Finally, the absorbance was measured at 535 nm and the MDA concentration was determined using standard curve.

### 2.4. Histopathological Procedures

The left kidney tissues were fixed in formalin 10% and embedded in paraffin for hematoxylin and eosin histopathological staining. Kidney tissue damage score (KTDS) was explained from 1 to 4 and presence of acute tubular damage such as tubular dilation and simplification, tubular cell swelling and necrosis, tubular casts, and intraluminal cell debris with inflammatory cell infiltration was considered. Score of zero was assigned to normal tissue.

### 2.5. Statistical Analysis

Data were presented as mean ± SEM. BUN, Cr, MDA levels, kidney weight (KW), uterus weight (UW), and body weight change (ΔBW) were compared by independent *t*-test analysis between control and GM groups in each gender. In addition, ANOVA analysis followed by LSD was used to compare the mentioned parameters among other groups. Kruskal-Wallis and Mann-Whitney tests were employed to compare KTDS. *P* values < 0.05 were considered statistically significant.

## 3. Results

### 3.1. Effect of ES on GM Induced Nephrotoxicity in Female

**Figure 1:**
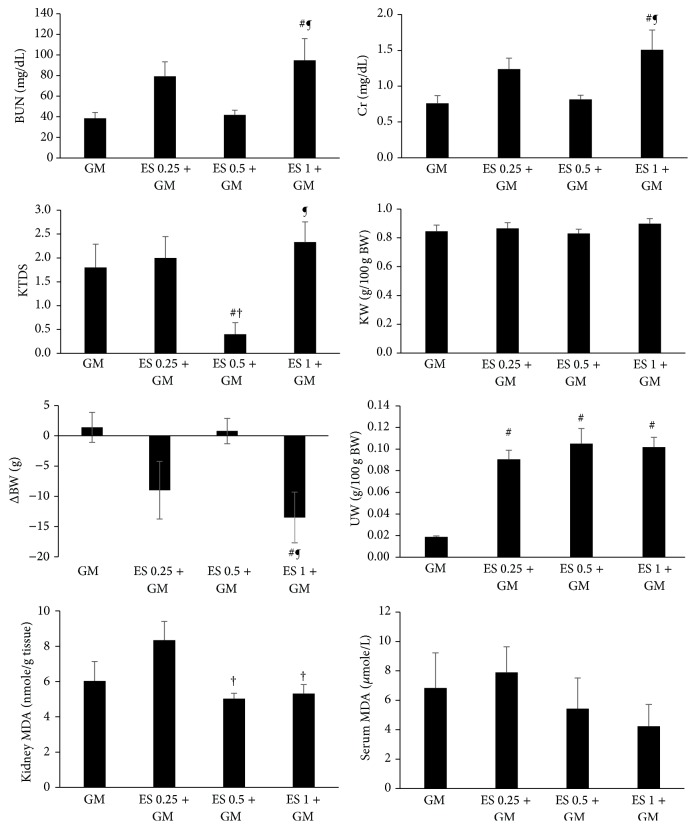
The effect of estradiol (ES) 0.25, 0.5, and 1 mg/kg on serum levels of blood urea nitrogen (BUN), creatinine (Cr), malondialdehyde (MDA), kidney level of MDA, kidney tissue damage score (KTDS), body weight change (ΔBW), kidney weight (KW), and uterus weight (UW) in female groups treated by gentamicin (GM). #, †, and ¶ indicate significant differences from GM, ES 0.25 + GM, and ES 0.5 + GM groups, respectively.

GM alone induced significant increment in KTDS and the serum levels of BUN and Cr in comparison with the control group (*P* < 0.05) (Table 2). Administration of ES (0.25 or 0.5 mg/kg) had no significant effect on the levels of BUN and Cr, although ES (0.5 mg/kg) ameliorated KTDS significantly (*P* < 0.05). On the other hand, ES (1 mg/kg) administration increased the levels of BUN and Cr in comparison with GM alone and ES (0.5 mg/kg) plus GM treated groups, significantly (*P* < 0.05). In addition ES (1 mg/kg) enhanced KTDS in comparison with ES (0.5 mg/kg) plus GM treated group (*P* < 0.05) (Figure 1). GM alone reduced BW significantly (*P* < 0.05) (Table 2), and administration of ES (0.25 or 1 mg/kg) intensified BW loss (Figure 1). UW was decreased by GM (Table 2) while as we expected ES (0.25, 0.5, or 1 mg/kg) increased UW significantly (*P* < 0.05) (Figure 1). There was no significant difference in KW between the groups (Table 2 and Figure 1). Significant changes in kidney level of MDA (*P* < 0.05) also were observed but such observation was not seen in serum level of MDA (Table 2 and Figure 1).

### 3.2. Effect of Pro with or without ES on GM Induced Nephrotoxicity in Female

**Figure 2:**
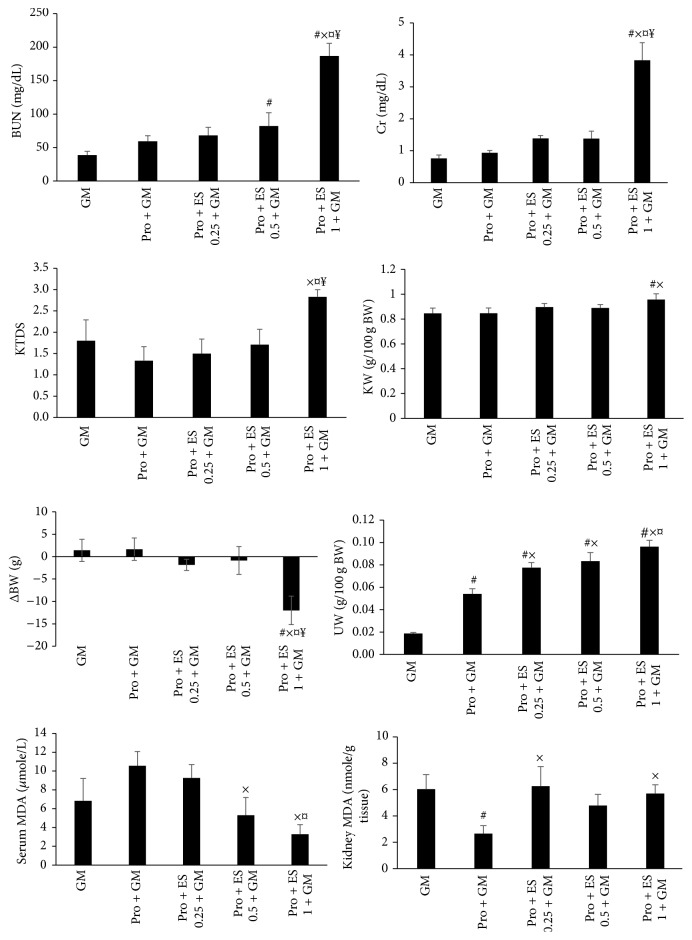
The effect of progesterone (Pro) without or accompanied with estradiol (ES) (0.25, 0.5, or 1 mg/kg) on the serum levels of blood urea nitrogen (BUN), creatinine (Cr), malondialdehyde (MDA), kidney level of MDA, kidney tissue damage score (KTDS), body weight change (ΔBW), kidney weight (KW), and uterus weight (UW) in female groups treated by gentamicin (GM). #, ×, *¤*,  and ¥ indicate significant differences from GM, Pro + GM, Pro + ES 0.25 + GM, and Pro + ES 0.5 + GM groups, respectively.

Pro with or without ES had no significant protective effects on BUN and Cr serum levels, KTDS, KW, and ΔBW. The combination of Pro and ES (0.5 mg/kg) increased the serum level of BUN in comparison with GM alone treated group significantly (*P* < 0.05). In addition, Pro accompanied with ES (1 mg/kg) enhanced BUN and Cr values as well as KW, KTDS, and BW loss significantly (*P* < 0.05). These observations showed negative effects of Pro plus ES on GM induced nephrotoxicity. As expected, both Pro alone and the combination of Pro and ES increased UW, significantly (*P* < 0.05). Also, significant difference in kidney and serum levels of MDA was observed among the groups (*P* < 0.05) (Figure 2).

### 3.3. Effect of TS and ES on GM Induced Nephrotoxicity in Male

**Figure 3:**
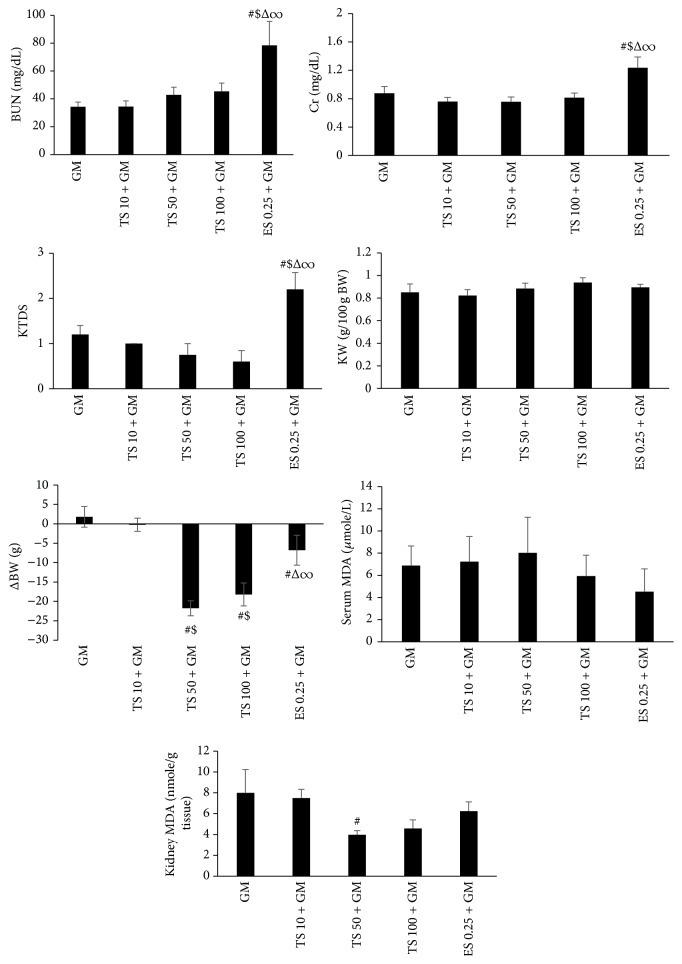
The effect of testosterone (TS) 10, 50, and 100 mg/kg and estradiol (ES) 0.25 mg/kg on serum levels of blood urea nitrogen (BUN), creatinine (Cr), malondialdehyde (MDA), kidney level of MDA, kidney tissue damage score (KTDS), body weight change (ΔBW), and kidney weight (KW) in male groups treated by gentamicin (GM). #, $, Δ, and ∞ indicate significant differences from GM, TS 10 + GM, TS 50 + GM, and TS 100 + GM groups, respectively.

GM alone increased the serum levels of BUN and Cr as well as KTDS significantly (*P* < 0.05) (Table 2) while TS administration had no effect on these parameters. However, ES (0.25 mg/kg) administration intensified the mentioned parameters significantly (*P* < 0.05) (Figure 3). GM decreased BW insignificantly when compared with control group (Table 2), but administration of TS (50 or 100 mg/kg) and ES (0.25 mg/kg) increased BW loss significantly (*P* < 0.05) (Figure 3). No significant difference was observed in KW (Table 2 and Figure 3). Also, GM alone changed serum and kidney levels of MDA in comparison with control group, significantly (*P* < 0.05) (Table 2). On the other hand, TS (50 mg/kg) decreased kidney MDA level significantly when compared with GM alone treated group (*P* < 0.05), but administration of TS and ES (0.25 mg/kg) induced no significant difference in serum level of MDA (Figure 3).

**Figure 4:**
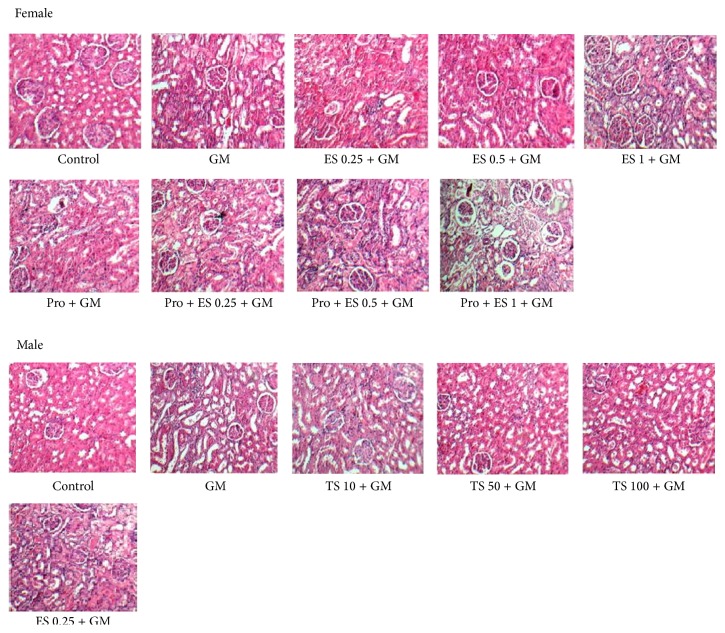
The kidney tissue images (100x) in all experimental groups.

The kidney tissue images for all the groups of experiment are shown in Figure 4.

## 4. Discussion

The objective of this study was to find whether administration of Pro and ES either alone or together could ameliorate nephrotoxicity induced by GM in ovariectomized rats. In addition, the effect of TS and ES on nephrotoxicity induced by GM in castrated rats was investigated. Our data indicated that GM induced nephrotoxicity in both male and female rats. In agreement with us other studies showed that GM increased BUN and Cr serum levels, KTDS, and KW [5, 26]. GM damages renal tubules especially proximal convoluted tubules [27] and induces apoptosis [8]. Also, other studies showed that GM reduced BW [26, 28] by reducing appetite and food consumption [29]. In addition, GM increased kidney level of MDA in both genders. GM induces oxidative stress [27] and decreases renal activity of antioxidant enzymes [28]. Also as unexpected data, this study showed that GM reduced serum level of MDA in male. Likewise, we observed that GM declined UW while this result was reported by the other study [30].

In agreement with us Ali et al. reported that TS administration did not affect renal histology and BUN and Cr values in castrated rats treated by GM [21]. Although one study showed that TS (10 mg/kg) ameliorated nephrotoxicity induced by CP such observation was not seen for TS 50 or 100 mg/kg [20]. Besides protective effect of TS was observed in renal failure induced by kidney ischemia-reperfusion in male rat [31], but we did not achieve significant results for TS. On the other hand, administration of ES (0.25 mg/kg) intensified nephrotoxicity induced by GM in male gender and other studies documented damaging effects of ES in male gender [32, 33]. ES also exacerbated renal failure induced by GM in intact male rat [23]. These observations indicated that ES has harmful effects on nephrotoxicity induced by GM in male gender.

TS (50 mg/kg) reduced kidney level of MDA. However, it was documented that special doses of TS had cytoprotective effects and could decrease lipid peroxidation [34] and reduce oxidative stress by androgen receptor-independent pathway [35].

Our finding indicated that ES (0.5 mg/kg) without Pro ameliorated renal damage induced by GM in female gender. In agreement with us, Ali et al. found that administration of ES (80 *μ*g/kg) ameliorated Cr and urea values in ovariectomized rats treated by GM [21]. It was reported that ES (500 *μ*g/kg) attenuated renal failure induced by kidney ischemia-reperfusion in female [33]. Also replacement of ES decreased proteinuria and glomerular injury in the remnant rat's kidney [25]. In addition, ES could ameliorate albuminuria and structural changes related to diabetes in female rat [36]. Furthermore, the combination of Pro and ES (0.5 mg/kg) had no protective effects on nephrotoxicity induced by GM; even it increased serum level of BUN. It seems that, at the presence of Pro, ES (0.5 mg/kg) could not exhibit its nephroprotective effects on toxicity induced by GM. It was documented that administration of ES restored Cr clearance and attenuated renal injury while the combination of ES and Pro had no effect on kidney damage and tended to decrease protective effects of ES on urinary excretion of protein and serum level of Cr [25]. One study also explained inhibitory effects of Pro on protective properties of ES [37]. Pro tended to decrease positive effects of ES on mediators expression of tissue fibrosis [25]. Our study indicated that not only did Pro and ES (0.25 or 1 mg/kg) either alone or together have no effect on nephrotoxicity induced by GM, but also both ES (1 mg/kg) alone and the combination of Pro and ES (1 mg/kg) exacerbated it. It is demonstrated that different doses of ES had no nephron-protective effect against nephrotoxicity induced by CP [19]; even ES administration reduced or reversed beneficial properties of supplementations such as erythropoietin [18], losartan, and vitamins E and C [17]. We observed destructive effects of high dose of ES (without or accompanied with Pro) on renal failure induced by GM. Meng et al. reported that high dose of ES induced negative effects on kidney function and histology in female mice and increased serum level of Cr, urine volume, and urinary excretion of protein and decreased Cr clearance [38]. On the other hand, Antus et al. showed that the combination of ES and Pro did not affect renal failure in nephrectomy model [25]. It seems that in our study the presence or absence of Pro accompanied with ES (0.25 or 1 mg/kg) did not impress renal failure induced by GM. Our previous study showed that both Pro (10 mg/kg) alone and the combination of ES and Pro ameliorated nephrotoxicity induced by CP [39]. Perhaps ES and Pro have different responses against various nephrotoxins. Results of this study showed that ES (0.5 or 1 mg/kg) without Pro reduced kidney (significantly) and serum level (insignificantly) of MDA. It was reported that very low dose of ES could not act as antioxidant agent [40] and we observed antioxidant properties of ES in high doses. Also Pro without ES reduced kidney level of MDA and Pro accompanied with ES (0.5 or 1 mg/kg) attenuated serum level of MDA. It is documented that Pro and ES have antioxidant properties [41, 42], although Pro accompanied with ES (0.25 or 1 mg/kg) increased kidney level of MDA in animals treated by GM. This observation was confirmed by the other study [39].

As expected, administration of different doses of ES without Pro increased UW which was confirmed by others [17–19, 33, 43]. Also Pro without or accompanied with different doses of ES enhanced UW. Ghasemi et al. showed that the combination of ES and Pro increased UW [39]. ES induces uterine growth and Pro has uterotrophic effects on uterus [44].

Present study demonstrated that both ES (1 mg/kg) alone and the combination of ES (1 mg/kg) and Pro induced body weight loss. Other study showed that the combination of these hormones prevented body weight gain by increasing lipid oxidation [45]. In addition, administration of ES (0.25 mg/kg) or TS (50 or 100 mg/kg) accompanied with GM induced BW loss in male gender. Other studies showed that high dose of TS itself induced BW loss in male rat [20, 46] via decreasing appetite and body fat [47]. In addition, ES diminishes BW and percentage of body fat and reduces protein content and caloric intake in both male and female [48].

## 5. Conclusion

Administration of ES 0.5 mg/kg without Pro ameliorated kidney damage induced by GM in female gender and presence of Pro attenuated this protective effect. On the other hand, neither TS nor ES had any beneficial effect on nephrotoxicity induced by GM in male gender while ES aggravated it. It seems that applications of GM in patients with hormones levels disturbances should be limited to avoid GM induced side effect of nephrotoxicity.

## Figures and Tables

**Table 1:** The design of experimental groups.

Gender	Group	Group's name	*n*	Treatment
Female	1	Control	6	Sesame oil + saline
	2	GM	5	Sesame oil + GM
	3	ES 0.25 + GM	5	0.25 mg/kg/week ES in sesame oil + GM
	4	ES 0.5 + GM	5	0.5 mg/kg/week ES in sesame oil + GM
	5	ES 1 + GM	6	1 mg/kg/week ES in sesame oil + GM
	6	Pro + GM	6	10 mg/kg/week Pro in sesame oil + GM
	7	Pro + ES 0.25 + GM	6	(0.25 mg/kg/week ES + 10 mg/kg/week Pro) in sesame oil + GM
	8	Pro + ES 0.5 + GM	7	(0.5 mg/kg/week ES + 10 mg/kg/week Pro) in sesame oil + GM
	9	Pro + ES 1 + GM	6	(1 mg/kg/week ES + 10 mg/kg/week Pro) in sesame oil + GM

Male	10	Control	4	Sesame oil + saline
	11	GM	5	Sesame oil + GM
	12	TS 10 + GM	4	10 mg/kg/week TS in sesame oil + GM
	13	TS 50 + GM	4	50 mg/kg/week TS in sesame oil + GM
	14	TS 100 + GM	5	100 mg/kg/week TS in sesame oil + GM
	15	ES 0.25 + GM	5	0.25 mg/kg/week ES in sesame oil + GM

GM: gentamicin, ES: estradiol, Pro: progesterone, TS: testosterone, and *n*: number of animals.

**Table 2:** The effect of gentamicin (GM) on the serum levels of blood urea nitrogen (BUN), creatinine (Cr), malondialdehyde (MDA), kidney level of MDA, kidney tissue damage score (KTDS), body weight change (ΔBW), kidney weight (KW), and uterus weight (UW) in male and female rats.

Gender	Group	BUN (mg/dL)	Cr (mg/dL)	Serum MDA (*μ*mole/L)	Kidney MDA (nmole/g tissue)	KTDS	KW (g/100 g BW)	UW (g/100 g BW)	ΔBW (g)
Female	Control (group 1)	21.79 ± 1.07	0.52 ± 0.08	5.49 ± 1.87	2.77 ± 0.20	0.33 ± 0.21	0.76 ± 0.02	0.02 ± 0.00	8.66 ± 1.68
	GM (group 2)	38.57 ± 5.67^*∗*^	0.76 ± 0.10^*∗*^	6.83 ± 2.38	6.03 ± 1.10^*∗*^	1.8 ± 0.48^*∗*^	0.84 ± 0.04	0.01 ± 0.00^*∗*^	1.4 ± 2.48^*∗*^

Male	Control (group 10)	23.70 ± 2.39	0.59 ± 0.04	11.65 ± 1.20	2.12 ± 0.20	0.5 ± 0.28	0.74 ± 0.04	—	7.25 ± 6.36
	GM (group 11)	34.27 ± 3.43^*∗*^	0.87 ± 0.09^*∗*^	6.88 ± 1.76^*∗*^	7.99 ± 2.23^*∗*^	1.2 ± 0.2^*∗*^	0.85 ± 0.07	—	1.8 ± 2.65

*∗* indicates significant difference from control group in each gender (*P* < 0.05).
